# Tandem mass tag-based quantitative proteomics and targeted hormone analysis reveal the response to insect herbivory stress in Ginseng (*Panax ginseng*, L.)

**DOI:** 10.1371/journal.pone.0316032

**Published:** 2025-01-22

**Authors:** Haitao Li, Lixin Zhang, Xiang Han, Qi Zhang, Guangna Liu, Guofeng Zhang, Yurong Zhu, Hongyang Liu, Haowei Deng, Shuangli Liu, Guangsheng Xi

**Affiliations:** 1 Jilin Agricultural Science and Technology University, Jilin, P. R. China; 2 National & Local Joint Engineering Research Center for Ginseng Breeding and Application (Jilin), Jilin Agricultural University, Changchun, Jilin, P. R. China; 3 Wuzhou University, College of Food and Pharmaceutical Engineering, Guangxi, P. R. China; Universidade de Coimbra, PORTUGAL

## Abstract

Ginsenosides are the most important secondary metabolites of ginseng. Ginseng has developed certain insect resistance properties during the course of evolutionary environmental adaptation. However, the mechanism underlying the insect resistance of ginseng is poorly understood. To elucidate the insect resistance mechanism of ginseng, we performed stress experiments on ginseng inoculated with black chafer larvae. The contents of ginsenosides in the ginseng roots, stems and leaves were determined at 0, 72, 120 and 168 h after the inoculation of insects. The tandem-mass-tag technology was used to determine the protein phosphorylation sites. Plant hormones were analyzed by multiple reaction monitoring targeted metabolomics. The results showed that ginsenosides present in the stems and leaves were more responsive to insect herbivory treatment than those present in the roots. Through proteomics, we found that the expression of most of the differentially expressed proteins, including GAPC1, GAPC2, and CSD1, was downregulated by insect herbivory treatment, HSP81-3 expression was up-regulated under insect herbivory stress. Regarding plant hormones, abscisic acid (ABA), gibberellic acid, Typhasterol (TY), iopentene adenine (IP), Cytokinin Riboside (czR) and Thiamethasone (tZ) levels were increased by herbivory treatment. With the increase in herbivory treatment time, the levels of trans-Zeatin-riboside (tzR), Isopentenyl adenosine riboside (iPR), and indole-3-acetic acid (IAA) were increased after 168h. The levels of salicylic acid (SA), jasmonates (JA), cis-PODA, and JA-Ile were increased after 120h but decreased thereafter. Under stress conditions, the expression of many antioxidant-related proteins was down-regulated; however, HSP81-3 expression was up-regulated, indicating that the plants exhibited severe oxidative stress. In conclusion, HSP81-3 plays an important role in ABA-dependent regulations involved in response to insect herbivory stress in ginseng. GAPC1 and GAPC2 also participate in the process of anti-herbivory stress response in ginseng.

## Introduction

Terpenoids are important secondary metabolites of plants that help them resist the adverse biotic environment and their synthesis pathways are closely associated with the adaptive evolution of plants against pest invasion [1]. Ginsenoside is an important terpenoid compound and the main effective component of Panax ginseng. So far, 201 triterpenoid monomer saponins have been identified from almost about 40 species of ginseng [2]. These compounds have many pharmacological activities such as antidiabetic [3], anti-cancerous [4,5], and anti viral [6], anti-tumor [7,8], and anti-oxidant [9]. In the ecosystem, terpenoids can regulate etabolism [10] and the mechanisms underlying insect resistance and bacteriostasis [11], hinder the growth and development of plants, and play crucial role in allelopathy [12].

During evolution, ginseng has developed a variety of response mechanisms against insect pests. Therefore, the ecological function of ginseng during the course of evolutionary environmental adaptation should be investigated. However, the mechanism underlying the resistance of ginseng against insect pests is not completely known. A previous study showed that the total ginsenoside content in ginseng has a significant inhibitory effect on the growth of *italic font* larvae, and this effect was dependent on the concentration of ginsenoside [13]. Another study showed that plant hormones, proteins, and transcriptional activation of defense-associated genes were crucial regulatory factors in response to the stress induced by insect pests [14]. However, the defense responses of ginseng against pests are largely unknown. In the present study, tandem mass tag (TMT)-based quantitative proteomics and targeted hormone assays were performed to analyze the responses to pest invasion in ginseng plants. This study has theoretical significance for further exploring the quality formation mechanism of genuine medicinal materials and the evolutionary pathway of plant environmental adaptability.

Our previous studies have confirmed that ginsenosides exhibit a broad-spectrum insect resistance activity against Lepidoptera, Coleoptera, Homoptera, Orthoptera, and more than 15 insect species, in addition to showing a significant antifeedant effect, avoidance effect, and interference with larval growth and development [15,16]. Thus, in insects, ginsenosides can inhibit the activities of detoxification, metabolic and protective enzymes affect food utilization; decrease the nutritional index of fatty acids; inhibit experimental population parameters; and decrease fecundity [6,17]. Ginsenosides play a crucial role in the adaptive evolution of ginseng against pest invasion; however, the underlying mechanism is unclear. In this study, we speculated that when pests ingest ginseng roots, they affect the synthesis of ginsenosides present in the ingested ginseng roots, which enables the mechanism against pest invasion via the signal transduction pathway of plant hormones present in the ingested roots. We quantitatively analysis the levels of monomer saponin and plant hormones in the roots, stems, and leaves after the insects ingested ginseng roots at different times. Quantitative proteomics and targeted hormone analysis based on TMT were performed to analyze the response of ginseng plants to pest attacks. The results of this study provide a theoretical basis for further investigating the mechanism underlying genuine medicinal plant substances, such as ginsenosides, and the evolutionary environmental adaptation of plants.

## Materials and methods

### Plant materials and insect herbivory treatment

In this study, 4-years-old plants of Jilin ginseng cv. *Damaya* were selected. Ginseng was grown under controlled temperature (20 °C) and natural light in a glass-equipped greenhouse affiliated with the Medicine Planting Garden of Jilin Agricultural University (Jinlin, China, 43°66’N, 126°49’E).

The black chafer (*Holotrichia parallela Motsch*.), in third instar and weighing 0.6–0.8 g, was provided by the Institute of Plant Protection, Chinese Academy of Agricultural Sciences (Beijing, China, 39.9°23’N, 116.3°18’E) and raised in an incubator under suitable conditions (temperature: 20°C; humidity: 55%; light cycle: 16 L-8 D; light intensity: 20000 LX) in the laboratory of traditional Chinese medicine, Jilin Academy of agricultural science and technology (Jinlin, China, 43°66’N, 126°49’E). Grubs were fed with 0.5 g potato chips every day.

All the grubs were given a week of adaptive rearing, and then, two grubs and one ginseng were kept in a container with soil to simulate the natural environment. The time at which the grubs climbed the stems or leaves of ginseng was recorded.

### Experimental design and sample collection

A total of four groups were set up in this study. For the control group (Col), ginseng was eroded by the grubs of black chafer at 0h (i.e. grubs have not eaten ginseng). For the insect herbivory treatment groups, ginseng was eroded by the grubs of black chafer at 72, 120 and 168 h, which was marked as T1, T2 and T3, respectively. The experiment in each group was performed in triplicates.

The ginseng stems and leaves in each group were placed in a drying box at 50 °C for drying, and taken out after 24 h. The dried samples were then crushed in a pulverizer (2–3 min). The pulverized powder was separately loaded in sealed bags for numbering and was stored in a dry and ventilated place for later use.

Each sample was divided into two parts; one part was used for TMT proteome analysis, whereas the other was used for targeted plant hormones analyses.

### Determination of ginsenoside content

The contents of ginsenosides Rg1, Rb2, Rc, Rd and Re were separated and detected using an SPD-10AVP HPLC system (Shimadzu, Tokyo, Japan) equipped with HC-C18 column (150 mm × 2.8 mm, 3 μm) (Agilent Corporation, CA, USA). For each separated ginsenoside, 20 μL samples were used in the HPLC system. A standard curve (correlation coefficients ≥ 0.999) was then constructed to determine the ginsenoside concentration.

### Protein extraction, trypsin digestion, and TMT labeling

Ginseng root proteins were extracted using the phenol extraction method. Ginseng root samples were powdered using liquid nitrogen, and then mixed with a four-fold volume of phenol extraction buffer (containing 10 mM dithiothreitol, 1% protease inhibitors and 2 mM EDTA). The mixture was then centrifugated at 5500 g for 10 min at 4 °C. Next, a 5-fold volume of 0.1 M ammonium acetate methanol buffer (4:1, v/v, pH = 3) was added to the supernatant, and the solution was kept overnight to form a precipitate. The extracted protein precipitate was washed three times with methanol and acetone. The washed proteins were digested with trypsin and then labeled with TMT tags. Then, an equal amount of samples was subjected to mass spectroscopy MS/MS analysis.

### Quantification of plant hormones

Quantification of plant hormones was performed as described previously [18]. Firstly, the freeze-dried plant tissues were homogenized at 4 °C in 80% acetonitrile containing 1% acetic acid. After 12 h, the mixture was centrifuged at 3,500 g for 15 min, and the supernatants, and hormone extracts, were collected. The hormone extracts were purified using solid-phase extraction columns (Waters, MA, USA). Finally, the concentration of hormones was detected using the LC-MS/MS system.

### LC-MS/MS analysis

LC-MS/MS analysis was performed on a Q Exactive HF mass spectrometer (Thermo Fisher, CA, USA), which was coupled with Easy nLC (Thermo Fisher, CA, USA). The prepared samples were then loaded into a reverse-phase C18 trap column (Chemicals Evaluation and Research Institute, Tokyo, Japan). Buffers A (0.1% Formic acid) and B (84% acetonitrile and 0.1% Formic acid) were used to separate the samples. The scanning parameters were as follows: MS resolution: m/z 200, 70,000; MS/MS: m/z 200, 17,500; normalized collision energy: 30 eV; underfill ratio: 0.1%.

### Bioinformatics analysis

Differentially expressed proteins (DEPs) were annotated and analyzed using the Gene Ontology (GO) and Kyoto Encyclopedia of Genes and Genomes (KEGG) database. Proteins with false discovery rate (FDR) <0.05 and log2FC > 1.2 or log2FC < 0.83 were considered DEPs. For pathway analysis, the DEPs were searched using the KEGG database by the KAAS program (http://www.genome.jp/kaas-bin/kaas_main) and FDR < 0.05 was considered statistically significant.

## Results

### Ginsenoside contents in different parts of ginseng

**Fig 1:**
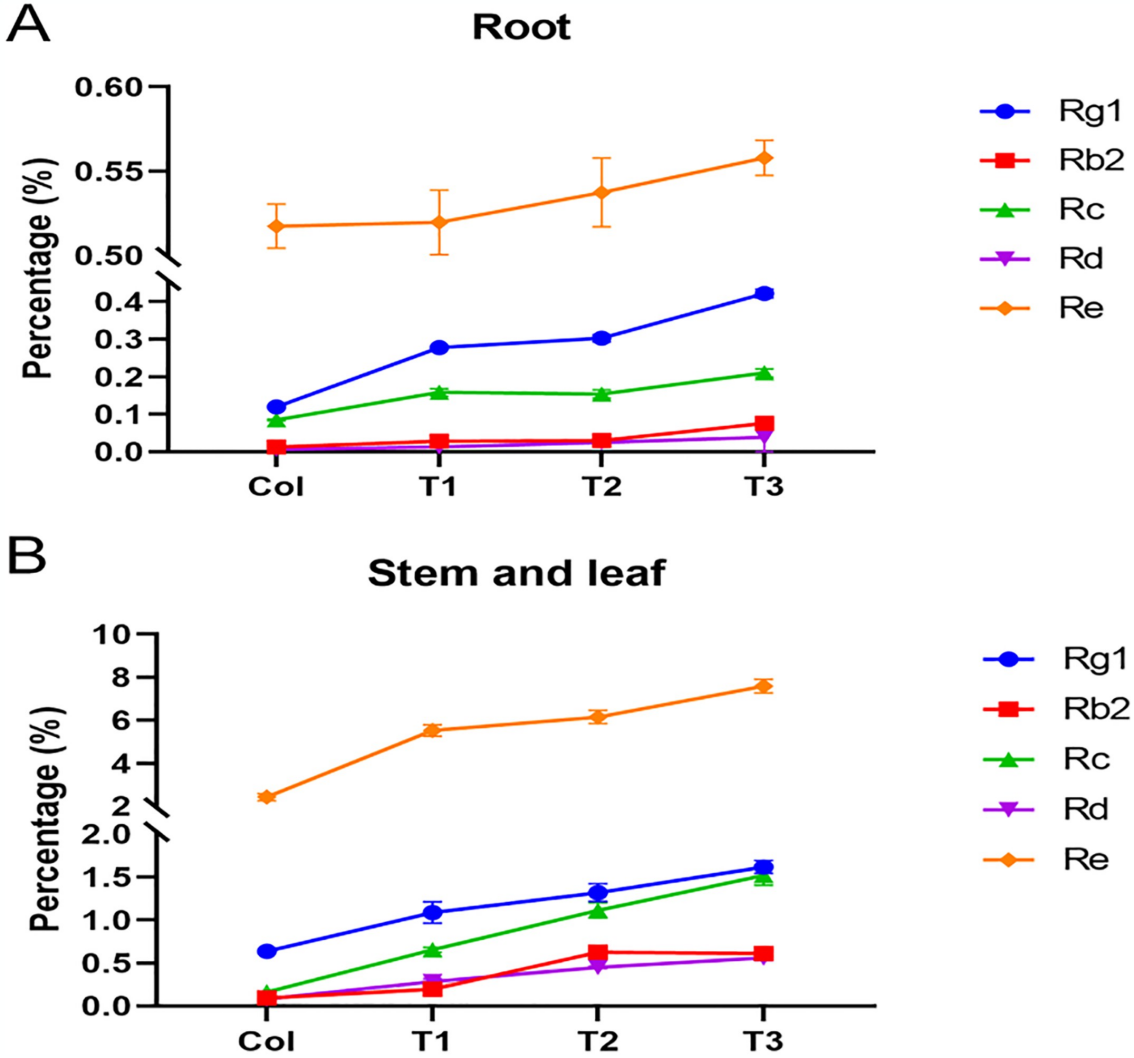
Effects of insect herbivory treatment on the content of ginsenosides (Rg1, Rb2, Rc, Rd, Re) in different parts of ginseng. (A) Ginseng ginsenosides content in root tissues. (B). Ginseng ginsenosides content in stems and leaf tissues. Ginseng was eroded by the grubs of black chafer for 0 h, 72 h, 120 h and 168 h was marked as Col, T1, T2 and T3, respectively.

All ginsenoside components, in the roots increased as the insect herbivory treatment time increased. The range of change of Rg1 was the largest, and T3 increased almost 4 times compared with that of the Col group (Fig 1A). We observed the different ginsenoside component patterns in the stems and leaf tissues. In the roots, the Rc content increased by almost 8 times while Rg1 and Re contents increased by about 3–4 times (Fig 1B), suggesting that ginsenosides in the stems and leaves were more responsive to insect herbivory treatment than in roots.

### Quantitative proteomics

A total of 302 phosphorylated proteins and peptides, and 1065 phosphorylation modification sites were identified, of which 210 proteins contained 266 quantifiable phosphorylated peptides and 681 quantifiable phosphorylation modification sites. Through the annotation results, the redundancy was removed, and 823 proteins were identified. To analyze the distribution of modification sites on proteins, the number of phosphorylation modification sites on all identified proteins was counted. The statistical results showed that 29.93% of the proteins contained at least two modification sites, and the Q1HGF1 protein contained up to 190 modification sites. Among all the phosphorylated peptides, the sites of phosphorylation on serine (S), threonine (T), and tyrosine (Y) were 99.05%, 8.64%, and 1.31%, respectively.

### Quantification of DEPs and enrichment analysis

**Fig 2:**
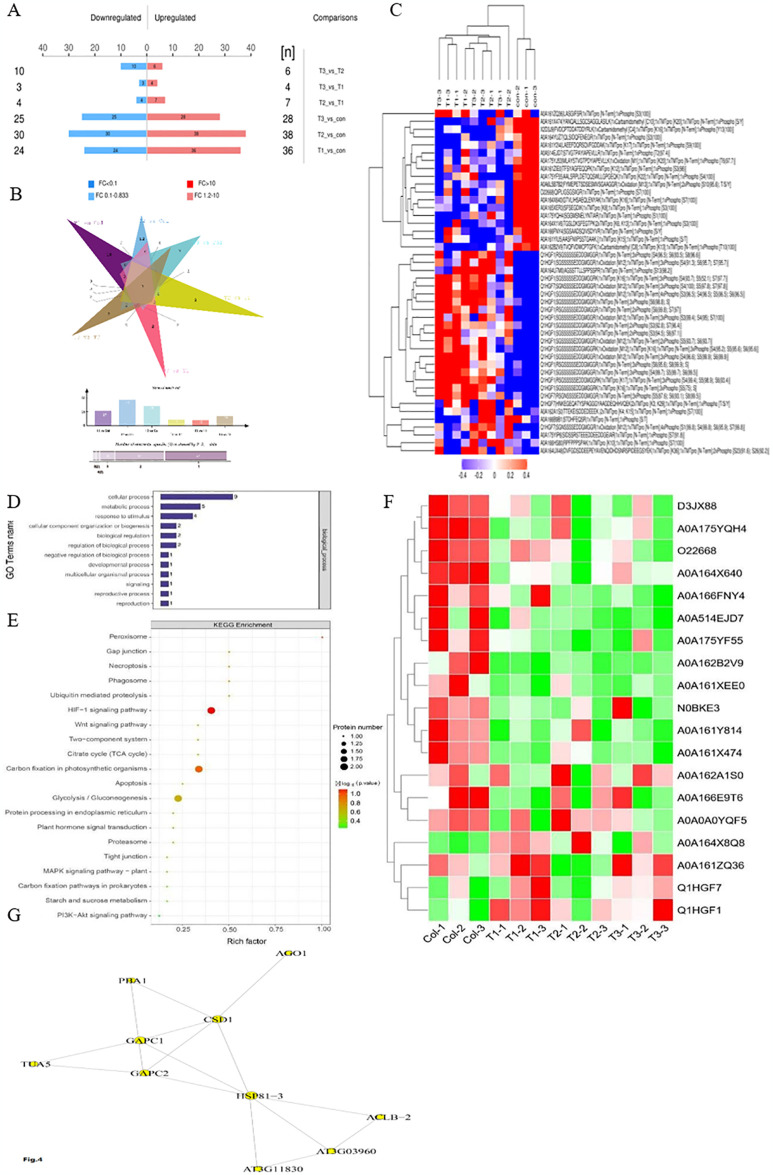
Quantification of differentially expressed proteins and enrichment analysis of DEPs. (A) The numbers of DEPs in different comparisons. Blue bar indicates the number of down-regulated proteins, and the red bar indicates the number of up-regulated proteins. (B) The Venn analysis of DEPs in different comparisons. (C) Expression of DEPs in all samples. Red indicates upregulation and blue indicates downregulation in the expression heatmap. Ginseng was eroded by the grubs of black chafer for 0 h, 72 h, 120 h and 168 h was marked as Col, T1, T2 and T3, respectively. (D) The GO enrichment analysis results. (E) The KEGG enrichment analysis results. (F). Expression of candidate DEPs. Red indicates upregulation and green indicates downregulation in the expression heatmap. Ginseng was eroded by the grubs of black chafer for 0 h, 72 h, 120 h and 168 h were marked as Col, T1, T2 and T3, respectively. (G) The interactive relationship of protein-protein network.

To quantify the DEPs in response to insect herbivory stress, the aforementioned proteome changes at different timepoints were detected. The numbers of DEPs that were up- or down-regulated between the six comparable groups is shown in Fig 2A. To further investigate the reversal effect of insect herbivory stress on protein expression in the ginseng roots, we constructed a Venn diagram and found that the number of DEPs was lower between different treatment groups, whereas the number of DEPs was higher between the treatment groups and Col (Fig 2B). A heatmap was drawn for the expression of all DEPs, and the results showed that the expression patterns among the different treatment groups were similar, whereas those between the treatment groups and Col were different (Fig 2C).

GO enrichment analysis of all DEPs showed that “response to stimulus” (GO:0050896), “cellular process” (GO:0009987) and “metabolic process” (GO:0008152) were the top three enriched GO terms (Fig 2D). KEGG enrichment analysis was performed based on all DEPs, and the results showed that the HIF-1 signaling pathway (ko04066), peroxisome (ko04146), carbon fixation in photosynthetic organisms (ko00710) and ubiquitin-mediated proteolysis (ko04120) were the significantly enriched pathways (Fig 2E). The enriched DEPs in GO and KEGG enrichment analyses included A0A0A0YQF5 (GAPC1), O22668 (CSD1), A0A161ZQ36 (UBC domain), A0A162B2V9 (TUA5), A0A162A1S0 (HATPase_c domain), A0A175YQH4 (ACLB-2), A0A175YF55 (Endoglucanase), A0A161X474 (Pectin acetylesterase), A0A166FNY4 (PBA1), A0A514EJD7 (SNF1-like protein kinase 3) and N0BKE3 (GAPC2). Among the DEPs screened in the GO and KEGG enrichment analyses, 19 were considered candidate DEPs after eliminating redundancy (Fig 2F).

Further analysis of the domain of candidate DEPs showed that all these mentioned DEPs contained 11 domains (Table 1). Three DEPs (A0A0A0YQF5, Q1HGF7, and N0BKE3) contained the same domain glyceraldehyde 3-phosphate dehydrogenase NAD binding domain.

**Table 1:** The domains contained in the DEPs.

DEPs	Domain_Name
O22668	Ubiquitin-conjugating enzyme
A0A161ZQ36	Ubiquitin-conjugating enzyme
A0A162B2V9	Tubulin C-terminal domain
A0A161XEE0	Tubulin C-terminal domain
A0A164X640	TCP-1/cpn60 chaperonin family
A0A514EJD7	Protein kinase domain
A0A166FNY4	Proteasome subunit
A0A164X8Q8	Proteasome subunit
A0A161X474	Pectinacetylesterase
A0A162A1S0	Hsp90 protein
A0A175YF55	Glycosyl hydrolase family 9
A0A0A0YQF5	Glyceraldehyde 3-phosphate dehydrogenase NAD binding domain
Q1HGF7	Glyceraldehyde 3-phosphate dehydrogenase NAD binding domain
N0BKE3	Glyceraldehyde 3-phosphate dehydrogenase NAD binding domain
Q1HGF1	Copper/zinc superoxide dismutase (SODC)
A0A175YQH4	Citrate synthase C-terminal domain

The interactive relationship of protein-protein was obtained through the STRING database, and then, the target protein-protein interaction (PPI) network map was drawn using Cytoscape 3.6.1. The PPI network showed that O22668 (CSD1), A0A162A1S0 (HSP81-3), A0A161YIU5, Q3LRW8, A0A0A0YQF5 (GAPC1), and N0BKE3 (GAPC2) were the core DEPs (Fig 2G).

### Targeted hormone analysis

**Fig 3:**
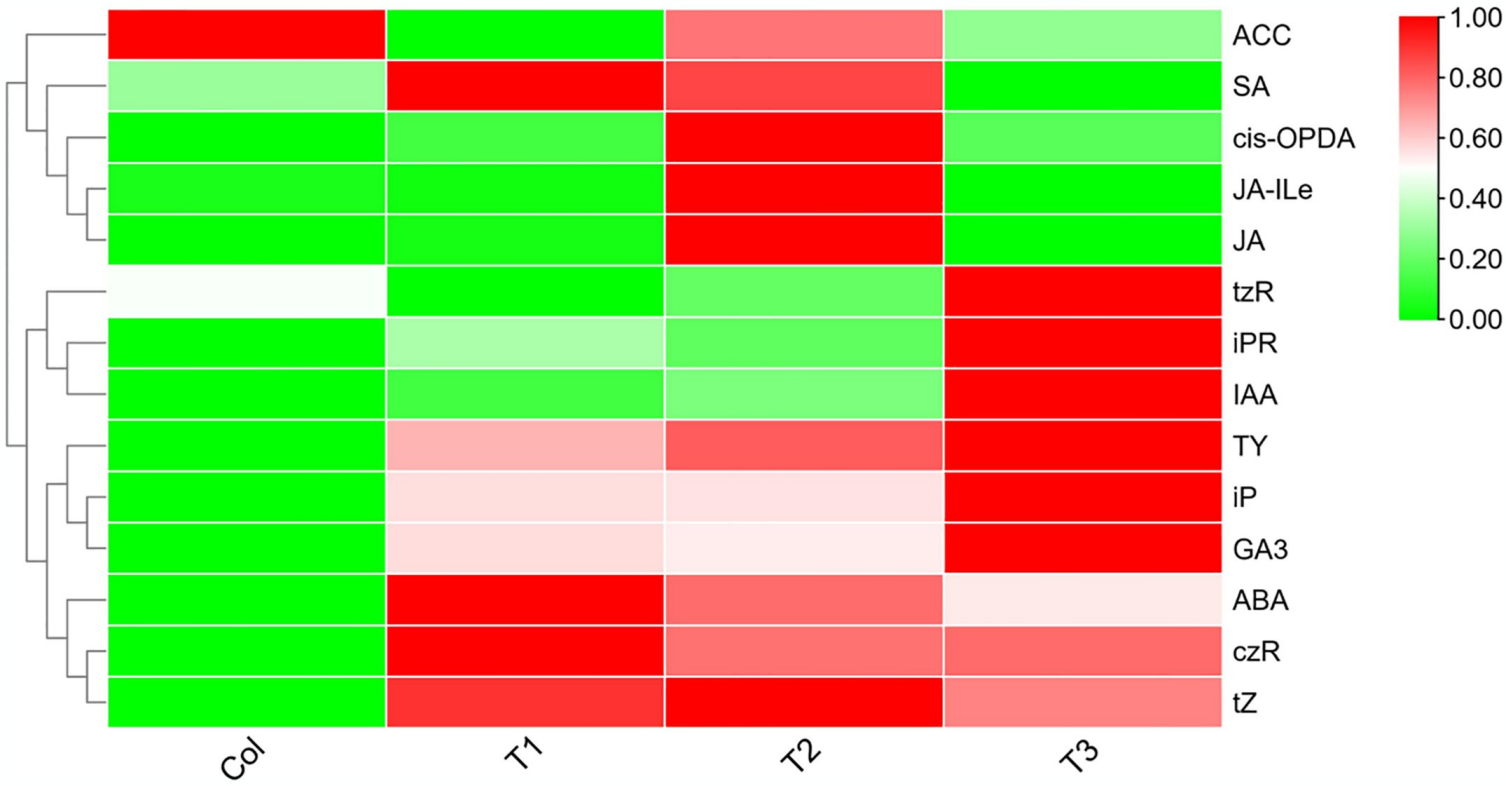
The content of targeted plants hormones. Red indicates upregulation and green indicates downregulation in the expression heatmap. Ginseng was eroded by the grubs of black chafer for 0 h, 72 h, 120 h and 168 h was marked as Col, T1, T2 and T3, respectively.

To determine the hormonal profile of the insect herbivory treatment group and Col, 21 plant hormones were analyzed in the methanolic extracts, belonging to the following classes: brassinosteroids, auxins, cytokinins, gibberellins, strigolactones, jasmonates (JA), salicylic acid (SA), and abscisic acid (ABA). Brassinosteroid lactone (BL), castasterone (CS), Dihydrozeaxanthin (DHZ), gibberellic acid (GA)1, GA4, GA7, and cis-Zeatin (cZ) were not detected in the plant extracts but these standards were detected in the mix of standards and matrices used as a positive control. This suggested that these hormones were not present in the extracts or under the limit of detection. Herbivory treatment increased the contents of ABA, GA3, Typhasterol (TY), Isopentene adenine (iP), Cytokinin Riboside (czR) and Thiamethasone (tZ). With the increament in herbivory treatment time, the contents of tzR, iPR and indole-3-acetic acid (IAA) increased after168h. The contents of SA, JA, cis-PODA and Jasmine ester L-isoleucine (JA-Ile) increased significantly after 120h and then decreased. However, ACC content decreased with the increased stress exposure time (Fig 3).

### Protein hormone interaction analysis

**Fig 4:**
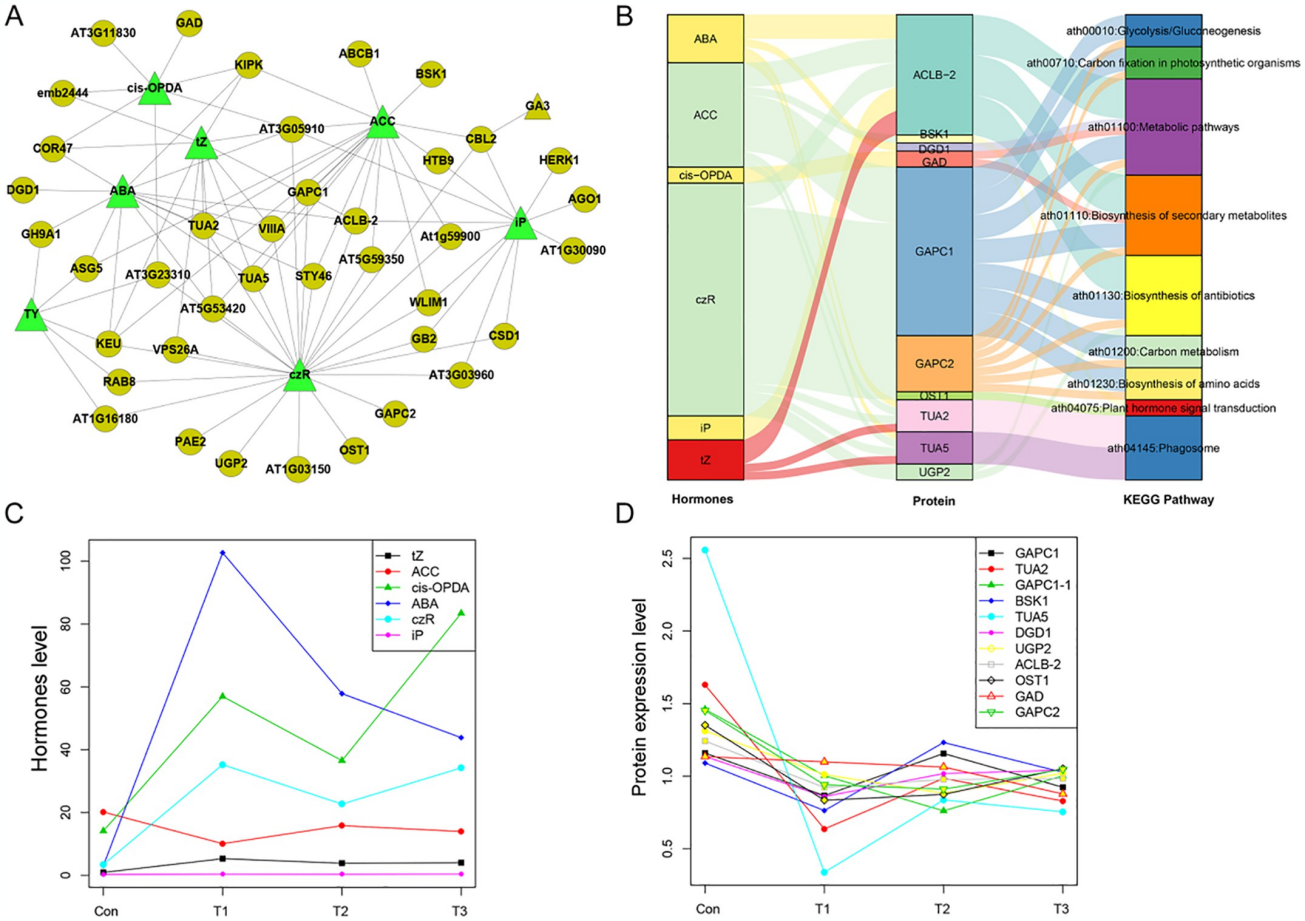
The interactive relationship of protein-hormone network and Sankey diagram direct shown the relationship between hormones, proteins and pathways. (A) The green triangles indicate hormones and the yellow circles indicates differential expressed proteins. (B) Sankey diagram between hormones, proteins and pathways. (C) and (D) the hormones and protein changes of dominate plant hormone and DEPs.

An interaction network was constructed based on the DEPs and plant hormones (Fig 4A). The results showed that ABA, czR, and iP were the major plant hormones interacting with multiple proteins. We also found that GAPC1 showed significant associations with ACC, tZ and czR. Furthermore, GAPC2 showed an association only with czR. The complex interactions showed that hormones and protein molecules are inextricably linked.

Furthermore, the connection analysis of the hormone-protein-pathway relationship was performed on proteins that are involved in the KEGG signaling pathway and the hormones that are related to proteins. The results showed that ABA, ACC and crZ were the dominant plant hormones, and ACLB, CAPC1, and CAPC2 were the dominant DEPs. These proteins and hormones are involved in multiple KEGG pathways, such as metabolic pathways, biosynthesis of secondary metabolites, and plant hormone signal transduction (Fig 4B). The level of tZ and ABA first increased and then decreased. The levels of cis-OPDA and czR increased at T1, then decreased at T2, and increased again at T3. Furthermore, the expression of most of the DEPs was downregulated after insect herbivory treatment (Fig 4C and 4D).

## Discussion

Unlike animals, plants lack an immune system; however, they have adapted some strategies against biotic stresses [19]. Plants have evolved complex defense systems that can respond quickly and effectively to herbivorous insects. Herbivory by insects has a profound impact on the primary metabolism of plants. To cope with insect herbivory, plants regulate their metabolite levels to reduce the damage caused by stress. There are differences in the responses of different plants to insect herbivory stress; for example, tobacco produces large amounts of nicotine, tomato increases the level of protease inhibitors, corn increases the content of benzoxazines, and Arabidopsis increases the level of thioglucoside [20–23]. In ginseng, we found that the level of ginsenosides significantly increased after insect feeding.

Earlier studies have shown that ginsenosides from ginseng stems and leaf have a It mainly regulates ABA-dependent or ABA-independent stress response pathways, and also participates in the biological stress response through the ABA biosynthesis pathway [11,24]. In a previous study, we found that ginsenosides decreased the profiles of free fatty acids (FAAs), and carbohydrates in the third instar larvae of *Ostrinia furnacalis* [25], which might be applicable to the present study as well. Furthermore, the bitter taste of ginsenosides makes them either insecticides or antifeedants for phytophagous insects [15]. Therefore, we believe that ginsenosides are the important metabolites of ginseng, and their remarkably increased levels in response to insect herbivory stress can reduce the adverse effect of insect pests on plants.

Oxidative radicals play a crucial role in plants under various stresses, including biotic stress such as insect infestation [26]. In the current study, HSP81-3 was the core DEP in the PPI analysis. HSPs exert a significant protective function in both biotic and abiotic stresses by controlling several cellular processes including chaperone activity [27,28]. A previous investigation has demonstrated that the increased accumulation (upregulation) of HSP81-3 can be a crucial defense strategy against salt stress [29]. Haq al. reported that HSPs play critical roles during biotic stress [30]. HSPs act as chaperones to maintain membrane stability [31], and they may use reactive oxygen species (ROS) as a signal molecule and scavenge them by positively regulating antioxidant enzymes [32–34], in addition to regulating plant growth and development under normal conditions [35–38]. Ubiquitin, a small HSP, degrades and disposes of denatured proteins. HSPs play a key role in stress response, which is a complex process; thus, they can be used in plant development. Furthermore, we discovered that GAPC1 and GAPC2 declined after the insect herbivory treatment. Reactive cysteine (Cys) residues in the active sites of GAPC1 and GAPC2 isoforms were susceptible to thiol modification and oxidation [39], suggesting the extent of oxidative damage in plants after insect herbivory stress, which is consistent with the result of a previous study [40]. Insect herbivory stress resulted in ROS accumulation and oxidative stress. ROS generated by metabolic processes can destroy cellular functions and have evolved to scavenge ROS. In the present study, the downregulation of CSD1 indicated that the ginseng plants had oxidative damage.

**Fig 5:**
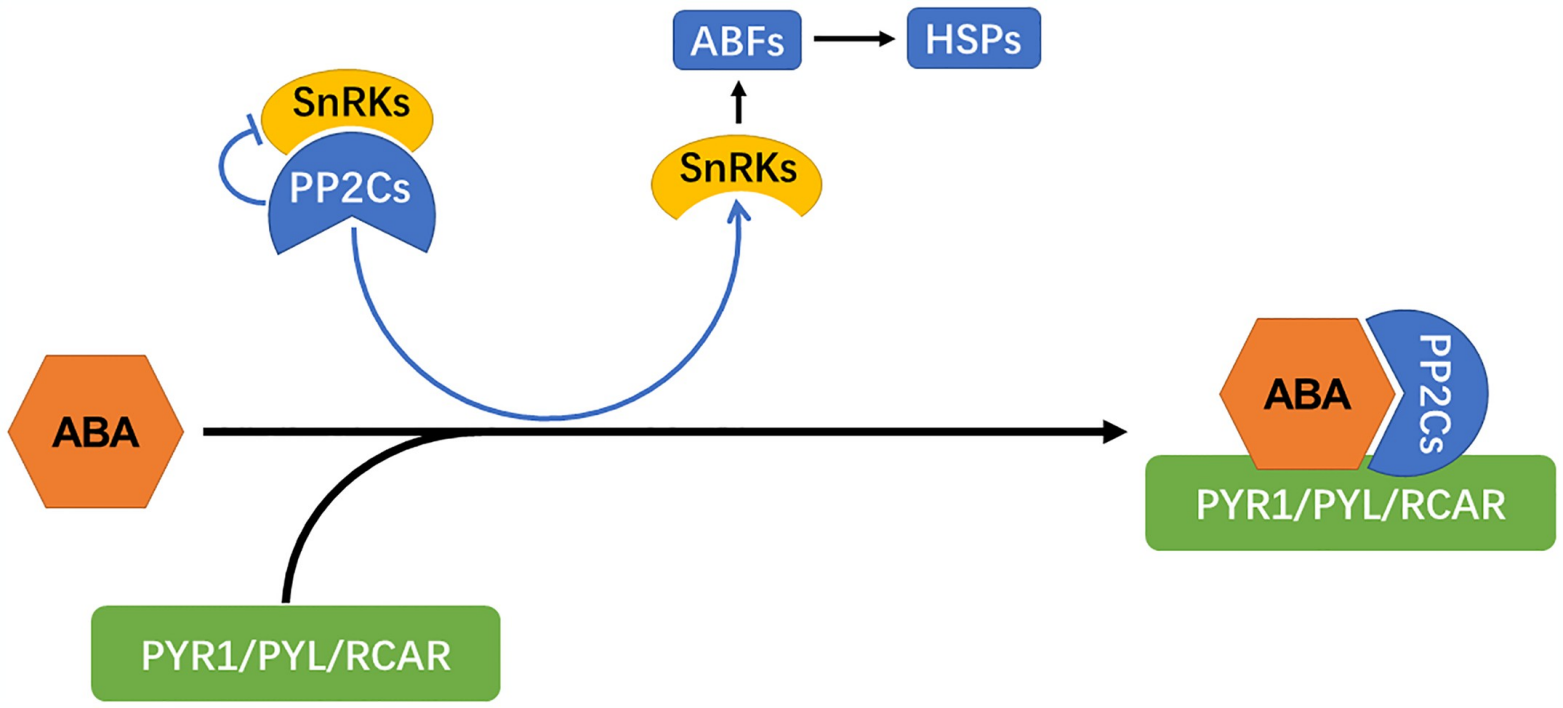
Schematic diagram of interaction between ABA signal pathway and HSPs.

Phytohormones are of vital importance for stimulating the defence response against insect herbivory. In this study, several signaling pathways, including those of ABA, GA, SA, IAA, czR, and tZ, probably orchestrated the induction of defense against insects. The levels of most of the mentioned phytohormones were increased under insect herbivory stress. ABA, GA, IAA, czR, and tZ are plant stress-related hormones [41–43]. For instance, the levels of ABA and IAA were augmented due to insect herbivory stress. The ABRE-binding protein/ABRE-binding factor family plays an significant role in environmental stress tolerance by regulating the diverse processes of plant development and metabolism [44]. In other plants, overexpression of DREB2A and DREB2C greatly induced the expression of HSP18.1-CI, HSP26.5-MII, and HSP70, enhancing heat and drought tolerance [45,46]. In the present study, HSP81-3 was highly induced, explaining the regulatory pathway of ginseng under insect herbivory stress (Fig 5).

H_2_O_2_ inhibits GAPC activity by oxidizing catalytic Cys residues within the enzyme [47]. The present results indicated a negative correlation between GAPC expression and ABA content. Guo et al. discovered that H_2_O_2_ enhances the interaction of GAPC with phospholipase D (PLDδ) by reducing the dissociation of GAPC—PLDδ binding. GAPC knockout weakened the ABA- or H_2_O_2_-induced production of phosphatidic acid (PA) in the cells, supporting the role of GAPCs in the H_2_O_2_-mediated activation of PLDδ [48]. A previous study demonstrated that the GAPC—PLDδ interaction mediates ROS signaling and enhances plant responsiveness to water deficiency [49]. This mechanism may also be applicable to the response to biotic stress in ginseng. When ginseng was exposed to insect herbivory stress, a large amount of ROS was produced in plant cells, which randomly induced oxidative stress. The levels of phosphoinositide phospholipase C (PLC), GAPC1, and GAPC2 decreased under insect herbivory stress in the present study. A previous study showed that the glyceraldehyde-3-phosphate dehydrogenase/GAPC pathway was targeted and modulated by PA from the PLC/diacylglycerol kinase pathway in response to a salt stress treatment [50]. Hence, we demonstrated that PLC, GAPC1, and GAPC2 play an important role in heat stress induced by insect herbivory stress.

## Conclusion

The present study revealed that ginseng exerted antifeeding effects on black chafer by increasing ginsenoside contents when exposed to insect herbivory stress. Under the insect herbivory stress, the levels of numerous antioxidant-related proteins were diminished; however, the level of HSP81-3 was elevated, suggesting that plants manifested severe oxidative stress. We further demonstrated that HSP81-3 was involved in ABA-mediated regulation in combination with the targeted hormone metabolome data. Furthermore, GAPCs play an important role in heat stress induced by insect herbivory stress.
